# Break-Induced Replication Requires DNA Damage-Induced Phosphorylation of Pif1 and Leads to Telomere Lengthening

**DOI:** 10.1371/journal.pgen.1004679

**Published:** 2014-10-16

**Authors:** Yulia Vasianovich, Lea A. Harrington, Svetlana Makovets

**Affiliations:** 1 Institute of Cell Biology, University of Edinburgh, Edinburgh, United Kingdom; 2 Institut de Recherche en Immunologie et en Cancérologie, Université de Montréal, Montréal, Québec, Canada; Chinese Academy of Sciences, China

## Abstract

Broken replication forks result in DNA breaks that are normally repaired via homologous recombination or break induced replication (BIR). Mild insufficiency in the replicative ligase Cdc9 in budding yeast *Saccharomyces cerevisiae* resulted in a population of cells with persistent DNA damage, most likely due to broken replication forks, constitutive activation of the DNA damage checkpoint and longer telomeres. This telomere lengthening required functional telomerase, the core DNA damage signaling cascade Mec1-Rad9-Rad53, and the components of the BIR repair pathway – Rad51, Rad52, Pol32, and Pif1. The Mec1-Rad53 induced phosphorylation of Pif1, previously found necessary for inhibition of telomerase at double strand breaks, was also important for the role of Pif1 in BIR and telomere elongation in *cdc9-1* cells. Two other mutants with impaired DNA replication, *cdc44-5* and *rrm3Δ*, were similar to *cdc9-1*: their long telomere phenotype was dependent on the Pif1 phosphorylation locus. We propose a model whereby the passage of BIR forks through telomeres promotes telomerase activity and leads to telomere lengthening.

## Author Summary

Telomeres are the ends of linear eukaryotic chromosomes maintained by an enzyme called telomerase. Non-telomeric DNA ends are often generated as a result of broken replication forks and are usually repaired by break-induced replication (BIR) or homologous recombination to avoid genomic instability. However, telomerase can interfere with the repair by adding a new telomere to a broken DNA end, or the break can be ligated to a telomere, thereby inducing genome re-arrangements that are often found in human genetic disorders and cancer. To understand how cells avoid erroneous repair, we studied *cdc9-1* yeast mutant cells that generate broken replication forks with high frequency. We discovered that, in cells with DNA damage, a helicase called Pif1 is phosphorylated and this phosphorylation enables Pif1 not only to inhibit telomerase at broken DNA ends but also stimulate the break repair by BIR, which in turn leads to additional telomere lengthening. Thus, a new regulatory pathway stimulates accurate break repair by BIR and at the same time promotes telomerase activity at telomeres.

## Introduction

Replication is a major source of nuclear DNA damage under normal mitotic growth conditions, i.e. in the absence of drugs or irradiation [1]. Studies in both prokaryotes and eukaryotes suggest that replication fork movement rates are heterogeneous, with slower fork movement, fork pausing and breaks at “hard-to-replicate” regions of the genome [2]. Stalled or broken replication forks are recognized as sites of DNA damage and activate a DDR (DNA damage response). The recognition of DNA damage leads to activation of a signaling cascade: phosphorylation is used to transduce the signal from the sensor kinases ATR and ATM (Mec1 and Tel1, respectively, in budding yeast) to the adaptors (Rad9 and Mrc1 in yeast), and then to the effector kinases Chk1 and Chk2 (Chk1 and Rad53/Dun1 in yeast), which in turn induce cell cycle arrest and DNA repair [3].

Broken replication forks generate a one-ended DSB that can be repaired by different mechanisms relying on DNA homology between the broken end and unbroken sister chromatid: homologous recombination, synthesis dependent strand annealing, or break-induced replication (BIR). During BIR, DSB processing by exo- and endonucleases generates a 3′ssDNA overhang that enables the homologous recombination machinery (Rad51/52/54/55/57) to invade a homologous sequence of the unbroken sister chromatid to re-establish the DNA synthesis [4]. Unlike conventional DNA replication, BIR employs a conservative replication mode [5], [6] and requires the Polδ subunit Pol32 and the 5′→3′ helicase Pif1 [7], [8].

Replication of telomeres, the ends of eukaryotic chromosomes, involves the enzyme telomerase. The core telomerase complex of *S. cerevisiae* includes an RNA component TLC1 [9] and the reverse transcriptase Est2 [10], [11]. Telomerase-dependent telomere synthesis is tightly coupled to conventional DNA replication and occurs in the S/G2 phase of the cell cycle [12], [13]. Telomerase can also add a telomere to a non-telomeric DNA end [14], [15], such as a DSB or a broken replication fork, thereby leading to a terminal deletion [16]. To prevent *de novo* telomere addition, the Pif1 helicase is phosphorylated in cells with DNA damage and the phosphorylated form of the protein inhibits telomerase at broken DNA ends [17]. Pif1 helicase is also a negative regulator of telomerase at telomeres under normal growth conditions [16]. Therefore, cells lacking Pif1 possess both longer telomeres and elevated frequencies of *de novo* telomere addition to DSBs [16], [18].

Although telomerase is inhibited at broken DNA ends through the phosphorylation of Pif1 by the DNA damage signaling machinery, as yet it remains unknown if any modulation of telomere synthesis in response to DNA damage takes place. Here we report that the DNA damage-induced phosphorylation of Pif1 is required not only for the inhibition of telomerase at DSBs but also for the function of Pif1 in BIR. In turn, activation of BIR in cells with DNA damage leads to telomere lengthening which may provide an additional layer of telomere protection against the DNA repair machinery [19]. Thus, the DNA damage-induced phosphorylation of Pif1 promotes potentially telomere-stabilizing telomere extension while repressing deleterious *de novo* telomere addition events at DNA breaks, thereby funneling the broken DNA into appropriate genome-preserving repair pathways.

## Results

### Telomerase-dependent telomere addition is increased in *cdc9-1* cells

During DNA replication, incomplete DNA ligation via partially abrogated ligase function may cause DNA discontinuities. In *S. cerevisiae*, *CDC9* encodes a replicative DNA ligase essential for yeast cell viability [20]. Like other temperature sensitive *cdc9* mutants, *cdc9-1* cells grow normally at 23°C but at 36°C arrest as large-budded cells with the nuclei at the bud necks [21]. The *cdc9-1* arrest is temporarily relieved in *cdc9-1 rad9Δ* cells, which at the non-permissive temperature undergo one or two cell divisions before losing viability [22].

**Figure 1:**
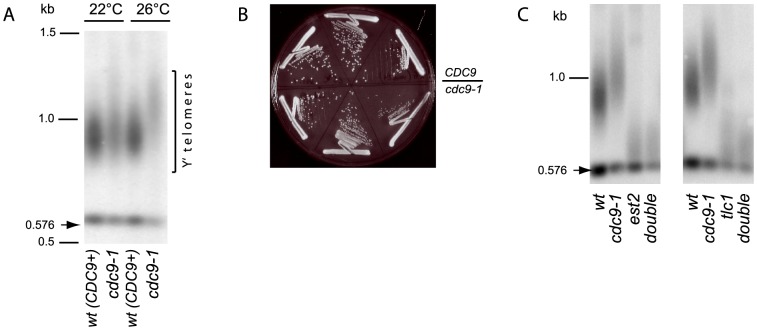
Telomerase-dependent telomere elongation is increased in *cdc9-1* cells at 26°C. (A) Telomere length in *CDC9* and *cdc9-1* cells at 22°C and 26°C assayed by Southern blotting (“teloblot”). (B) *CDC9* and *cdc9-1* colony growth at 26°C on rich medium. The photo was taken after 48 h incubation. (C) Telomere lengthening in response to *cdc9-1* requires functional telomerase. The teloblots are shown as sets of four samples representing four spore progeny from the same tetrad germinated at 22°C and grown for ∼40 generations (2 passages) at 26°C. DNA standards (in kbp) shown at left.

It has been reported previously that *cdc9-1* mutants have longer telomeres [23]. When grown at 22°C, the *cdc9-1* mutant strain possessed a bulk telomere length similar to *CDC9* cells (Figure 1A). However, when *cdc9-1* yeast were propagated at an intermediate temperature, 26°C, after about 80 generations the telomeres were distinctly and reproducibly elongated (mean telomere length 450 bp) compared to the isogenic *CDC9* strain (mean telomere length 350 bp) (Figure 1A). At this semi-permissive temperature (26°C), *cdc9-1* cells showed only a very mild growth defect phenotype and possessed a slightly smaller colony size (Figure 1B).

The telomere length phenotype in *cdc9-1* cells could have arisen from any of the following possibilities: (i) increased telomerase action, (ii) increased telomeric recombination, (iii) impaired telomere processing/shortening. To distinguish between these possibilities, we compared the dynamics of telomere length changes in response to *cdc9-1* in the presence and absence of telomerase. The double heterozygous diploids *CDC9/cdc9-1 EST2/est2Δ* and *CDC9/cdc9-1 TLC1/tlc1Δ* were sporulated and germinated spores grown into single colonies (about 20 generations) at 22°C. The four progeny spores from the tetrads were subsequently grown on plates at 26°C for a further ∼40 generations (two streaks) for telomere length analysis. As expected, in the presence of telomerase, the *cdc9-1* cells possessed longer telomeres than *CDC9* cells (Figure 1C). However, in cells lacking Est2 or TLC1, the *cdc9-1* mutation had no effect on telomere length: both *CDC9* and *cdc9-1* cells lacking either essential telomerase component showed similar rates of telomere shortening. Thus, the telomere lengthening in response to *cdc9-1* was dependent on functional telomerase and was not a result of impaired telomere erosion or end processing.

### Constitutive activation of the DNA damage signaling leads to telomere lengthening in *cdc9-1* cells

**Figure 2:**
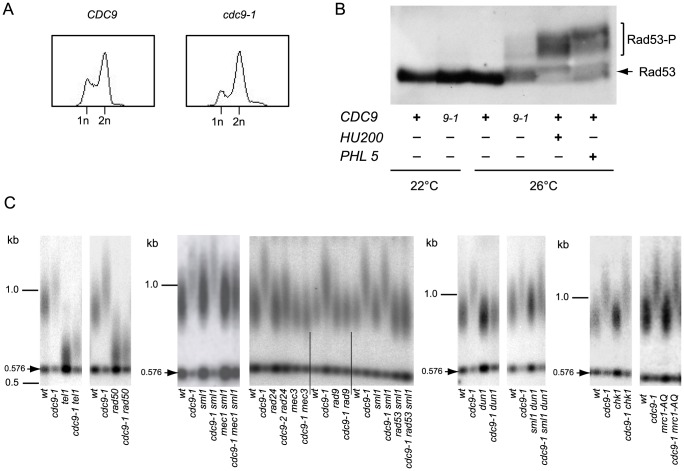
Activation of the DNA damage checkpoint occurs in *cdc9-1* cells at 26°C and is required for their telomere length phenotype. (A) The *cdc9-1* mutation leads to accumulation of late S/G2 cells at 26°C. (B) Rad53 activation in response to *cdc9-1* at 26°C, or after treatment with either hydroxyurea (200 mM, *HU200*), or Phleomycin (5 µg/ml, *PHL5*) for 2 h at 26°C. (C) Epistasis analysis of telomere lengthening (assayed by teloblot) in response to *cdc9-1* involving mutations in genes for different components of the DNA damage signaling network. Telomere length analyses by teloblots which are shown either as sets of four samples representing four spore progeny from the same tetrad or sets of six spores from different tetrads but the same parental strain (for *mec1* and *rad53*), with telomere length equilibrated at 26°C. DNA standards (in kbp) shown at left.

FACS analysis indicated that non-synchronized populations of haploid *cdc9-1* yeast were enriched for cells with 2n DNA content (Figure 2A), suggestive of a cell cycle progression delay in late S-phase or G2, perhaps due to checkpoint activation. In response to DNA damage, a central component of the DNA damage signaling pathway, Rad53, is activated by phosphorylation [24]. In *cdc9-1* cells at 26°C, but not 22°C, a shift in Rad53 mobility upon SDS-PAGE characteristic of Rad53 phosphorylation in response to DNA damage was observed (Figure 2B). Therefore, at the semi-permissive temperature the *cdc9-1* mutation leads to DDR activation.

We tested the genetic dependency of the telomere lengthening in *cdc9-1* cells at 26°C on known DNA damage signaling network components. Diploid strains heterozygous for both *CDC9/cdc9-1* and each gene of interest were sporulated and the spores germinated at 22°C. In the case of *MEC1* or *RAD53*, the cells were also heterozygous for *SML1/sml1Δ* in order to suppress the known lethality of *mec1Δ* or *rad53Δ* with *sml1Δ* in the haploid progeny [25]. Progeny with different genotypes were propagated at 26°C for 4 re-streaks (∼80 generations) to allow telomere length to reach the stable length characteristic of each genotype.

Telomere elongation in *cdc9-1* cells was retained in a *tel1Δ* or *chk1Δ* background, in the replication checkpoint deficient mutant *mrc1-AQ* [26] and in an *sml1Δ dun1Δ* background where *sml1Δ* suppresses the effect of *dun1Δ* on S-phase progression [27]. In contrast, the telomere lengthening in *cdc9-1* cells was dependent on *RAD50*, *MEC3*, *RAD24* (to some extent), *MEC1*, *RAD9*, and *RAD53* (Figure 2C).

Cdc17 is a catalytic subunit of the DNA polymerase Polα that plays a pivotal role in the coordination of telomere synthesis and conventional DNA replication [28]. Like *cdc9-1*, a temperature sensitive mutation, *cdc17-1*, results in longer telomeres [23], [29]. However, *cdc17-1* did not exhibit constitutive activation of DDR and the *cdc17-1* induced telomere lengthening was independent of the DNA damage checkpoint (Figure S1). Therefore, we conclude that the telomere elongation phenotype of *cdc9-1* at 26°C is not a defect related to the coordination of lagging strand synthesis at telomeres, but rather depends on activation of the central DNA damage checkpoint network (*RAD50*, *MEC3*, *RAD24*, *MEC1*, *RAD9*, and *RAD53*) but not on *TEL1*, *MRC1*, *DUN1* or *CHK1*. Thus, the effect of *cdc9-1* on telomere synthesis is likely to be indirect, via activation of the DNA damage signaling which then leads to telomere lengthening.

### Phosphorylation of nPif1 via DNA damage signaling machinery is required for the *cdc9-1* induced telomere lengthening

We used a candidate approach to identify a potential link between the activation of the DNA damage response and telomere lengthening in *cdc9-1* cells based on (i) a previously reported role for the candidate proteins in telomere metabolism; (ii) the candidate proteins are regulated by the DNA damage signaling machinery. A set of candidates came from the report that activation of DDR leads to dynamic changes in telomere architecture [30]. The yeast telomeric chromatin components Rap1, Sir2/3/4, Rif1, and Rif2 (which regulate telomerase action on a telomere in *cis* [31]), and the Ku70/80 complex (which is required for telomerase localization in the nucleus and at the telomeres [32]) re-localize away from telomeres in a Rad9-dependent manner [30]. Such re-localization during DDR activation in *cdc9-1* could lead to longer telomeres. We analyzed the effect of the *cdc9-1* mutation on telomere length in cells deleted for *RIF1*, *RIF2*, *SIR2*, *SIR3*, *SIR4*, *YKU70*, or *YKU80*. The telomere length increase observed in *cdc9-1* cells did not depend on *RIF1* or *RIF2* but was moderately decreased in the absence of the Sir proteins, and no telomere elongation was observed in either *yku70* or *yku80* backgrounds (Figure S2). Therefore, the telomere elongation observed in *cdc9-1* cells is dependent on Ku70/80 and at least partially dependent on Sir proteins. The Ku70/80 dependence is in accord with the known requirement of Ku70/80 in telomerase recruitment [32], while the *SIR*-dependence could reflect known roles in telomere architecture and telomerase regulation in *cis*, or as yet unexplored role(s) in BIR-dependent telomere replication.

**Figure 3:**
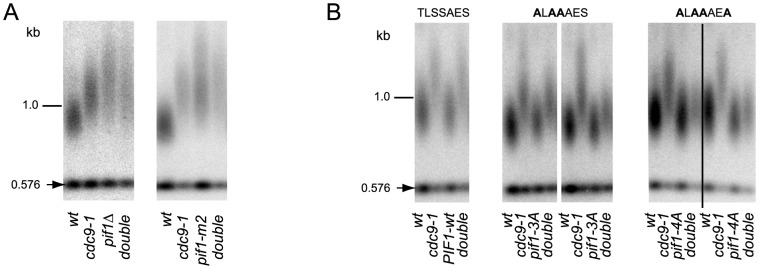
Telomere elongation in *cdc9-1* is dependent on the presence of nPif1 and its phosphorylation locus. Telomere length analyses by teloblots which are shown as sets of four samples representing four spore progeny from the same tetrad, with telomere length equilibrated at 26°C. (A) Analysis of *cdc9-1* induced telomere elongation in *pif1Δ* and *pif1-m2* (loss of nPif1) backgrounds. (B) Telomere length analysis of *CDC9* and *cdc9-1* in *pif1* TLSSAES mutant backgrounds. The mutated TLSSAES loci with substituted amino acids in bold are shown above the blot images. DNA standards (in kbp) shown at left.

DNA damage-induced signaling is also known to regulate a nuclear form of the Pif1 helicase [17], a well characterized inhibitor of telomerase at both telomeres and DNA breaks [16]. Phosphorylation of nuclear Pif1 (nPif1) in response to DSBs is required for telomerase inhibition at broken DNA ends [17]. To query if nPif1 and its phosphorylation during the DDR were responsible for the telomere lengthening in *cdc9-1* cells, we combined *cdc9-1* with *pif1Δ, pif1-m2* (nPif1 null [16]), or the previously reported *PIF1* alleles *pif1-3A* and *pif1-4A* that abrogate Mec1-Rad53-dependent phosphorylation of the peptide TLSSAES [17]. Like *cdc9-1*, *pif1Δ* and *pif1-m2* have longer telomeres but no additive telomere lengthening was observed in either *cdc9-1 pif1Δ* or *cdc9-1 pif1-m2* (Figure 3A). Furthermore, both *pif1-3A* and *pif1-4A* mutations alleviated the *cdc9-1* induced telomere lengthening, with slightly more pronounced effect in a *pif1-4A* background compared with *pif1-3A* (Figure 3B), suggesting a possibility of nPif1 phosphorylation playing a role in telomere elongation in *cdc9-1* cells. Thus, the telomere lengthening in *cdc9-1* cells is genetically dependent on the presence of the nuclear form of Pif1 and specifically on the phosphorylation of TLSSAES.

**Figure 4:**
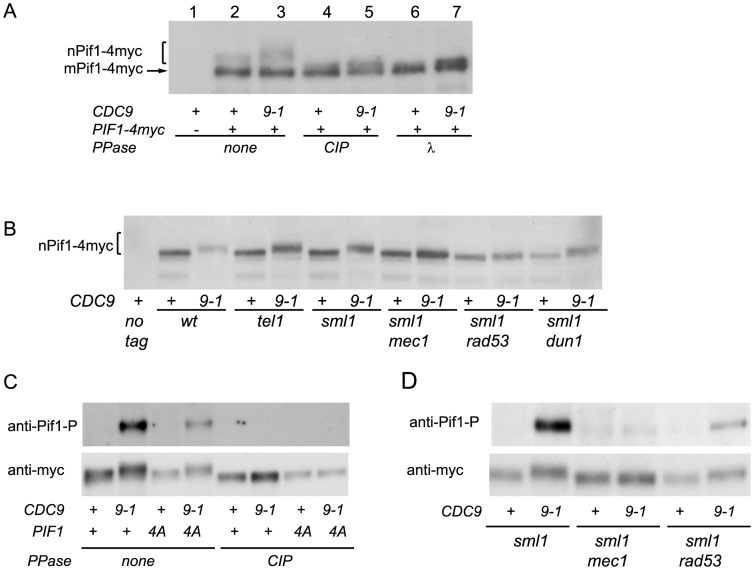
nPif1 is phosphorylated in *cdc9-1* cells and this phosphorylation is required for telomere elongation. (A) nPif1 is phosphorylated in *cdc9-1* cells. Expression of *PIF1-4myc* results in two polypeptides. The species with the faster gel mobility corresponds to the mitochondrial Pif1 (mPif1) and the slower migrating protein is nuclear Pif1 (nPif1). Pif1-4myc was immunoprecipitated (IPed) from *CDC9* and *cdc9-1* cells as well as from the control *CDC9 PIF1* (no tag) strains. Immunoprecipitated material was treated with either Calf Intestinal Phosphatase (CIP) or λ phosphatase and compared to mock-treated samples (lanes 2 & 3) and analyzed by SDS-PAGE. (B) nPif1 phosphorylation in response to *cdc9-1* depends on *MEC1* and *RAD53*. Note, in this panel and all the experiments below only nuclear Pif1 was tagged with 4myc (the *pif1-m2::URA3-pif1-m1-4myc* allele, see Table S1) and therefore the mitochondrial Pif1 was no longer visible on western blots. (C) TLSSAES phosphorylation in response to *cdc9-1*. Note that the anti-P-Pif1 antibody has weak cross-reactivity with another DNA damage induced phosphorylation site on Pif1 (lane 4: *cdc9-1 pif1-4A*). However, this cross-reactivity is unrelated to TLSSAES as there is a significant difference between *PIF1 cdc9-1* and *pif1-4A cdc9-1* (compare lanes 2 and 4) in the relative amount of the signal; see also panel D. (D) TLSSAES phosphorylation in response to *cdc9-1* is *MEC1-RAD53*-dependent. In panels (C–D), nPif1-4myc and nPif1-4A-4myc were immunoprecipitated using an anti-myc antibody (9E10) from cells with the genotypes as indicated below, then treated or mock-treated with CIP phosphatase, resolved on SDS PAGE, transferred onto PVDF membrane, probed using an affinity purified rabbit polyclonal antibody raised against VIDFYL(pT)LS(pS)AE (anti-P-Pif1, upper image on each panel) and then re-probed with 9E10 (anti-myc, lower image).

To test whether nPif1 was phosphorylated in *cdc9-1* cells, we immunoprecipitated Pif1-4myc from *CDC9* and *cdc9-1* cells and treated a third of each sample with either CIP or λ phosphatase and compared their mobility by SDS-PAGE (Figure 4A). Indeed, nPif1 migration was retarded to a greater extent in *cdc9-1* than in *CDC9* cells and phosphatase treatment eliminated this mobility difference. In fact, phosphatase treatment resulted in faster mobility nPif1 species in both *CDC9* and *cdc9-1* cells (Figure 4A: compare lane 2 to lanes 4 and 6). Together, these findings suggest that nPif1 possesses a basal level of phosphorylation in wild-type cells, and that additional phosphorylation events occur in response to *cdc9-1*. Consistent with the DDR-dependent modulation of nPif1 activity at DSBs, nPif1 phosphorylation in response to *cdc9-1* depended on *MEC1* and *RAD53* but not *TEL1* or *DUN1* (Figure 4B). To further probe nPif1phosphorylation, we used an antibody specific to the nPif1 phospho-regulatory locus (pT)LS(pS)AE (anti-P-Pif1 antibody) [17] and established its phosphatase-dependent recognition of nPif1 in *cdc9-1* (but not *CDC9*) cells that was largely abrogated by the *pif1-4A* mutation (Figure 4C, top panel). In *sml1Δ mec1Δ* and *sml1Δ rad53Δ* backgrounds, the *cdc9-1* dependent recognition of Pif1 with the anti-P-Pif1 antibody was considerably reduced (Figure 4D). Taken together, the data indicate that *cdc9-1* activates *MEC1-RAD53*-dependent phosphorylation of nPif1 on TLSSAES (as well as on other positions on Pif1), and that the resulting telomere lengthening minimally requires TLSSAES phosphorylation.

### Telomere lengthening in *cdc9*-1 requires functional components of the BIR pathway

**Figure 5:**
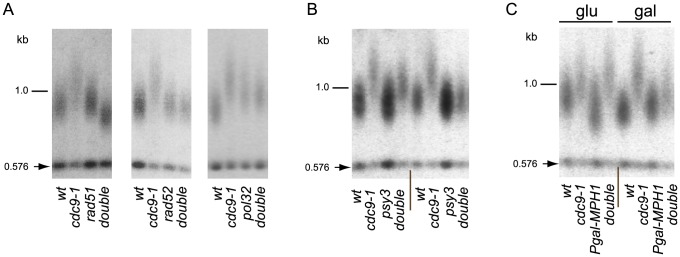
BIR is required for telomere elongation in *cdc9-1* cells. Telomere length analyses by teloblots, shown as sets of four samples representing four spore progeny from the same tetrad grown at 26°C. (A) Analysis of *cdc9-1* induced telomere elongation in the absence of the essential components of BIR - Rad51, Rad52, or Pol32; (B) Telomere elongation in response to *cdc9-1* is alleviated in a *psy3* background. Notice that variability in telomere length of *cdc9 psy3* mutants was observed and therefore two tetrads representing most extreme examples are shown. (C) Over-expression of *MPH1* from a galactose-inducible promoter in cells propagated on galactose suppresses telomere elongation in *cdc9-1* cells (see the set of 4 samples marked **gal** on the right). The four spore progeny from the same tetrad were passaged for ∼80 generations either on glucose (**glu**, the *GAL1* promoter is repressed) or on galactose (**gal**, the *GAL1* promoter is induced) before the DNA samples were prepared and analyzed for telomere length. DNA standards (in kbp) shown at left.

In addition to its roles in regulation of telomerase at telomeres and DSBs [16], [17], nPif1 also has been reported to have a role in BIR [8]. The *cdc9-1* mutation causes replicative ligase insufficiency which results in nicks as evidenced from accumulation of unligated nascent DNA strands [20], [33]. Passage of replication forks through these nicks would lead to broken replication forks [34] that would require homology-mediated repair, such as homologous recombination or BIR. To test if BIR had any role in telomere lengthening in *cdc9-1* cells, we combined *cdc9-1* with deletions in genes encoding the major components of BIR machinery, such as Rad51, Rad52, and Pol32. Indeed, we observed that *RAD51*, *RAD52*, and *POL32* were required for the *cdc9-1* induced telomere elongation (Figure 5A). Further, the *cdc9-1* induced accumulation of G2 cells was retained in *pif1-4A* and *pol32Δ* backgrounds (Figure S3) despite the absence of telomere lengthening (see Figures 3B and 5A), suggesting that *PIF1* and *POL32* are downstream of the DDR activation in the pathway leading to telomere elongation in *cdc9-1* cells. Because Pol32 and nPif1 are required specifically for BIR [7], [8] but not for homologous recombination, our data suggest that BIR plays a major role in telomere elongation in *cdc9-1* yeast.

To explore this hypothesis further we tested if the PCSS complex (Psy3/Csm2/Shu1/Shu2) and Mph1 helicase, known to regulate Rad51 filament formation and post-invasion steps in BIR respectively [35], [36], [37], had an effect on telomere length in *cdc9-1*. Deletion of *PSY3* partially suppressed telomere elongation in *cdc9-1* cells (Figure 5B), consistent with the previously reported role for the PCSS complex as a positive regulator of Rad51 filament assembly on a processed DSB [35], [36]. Mph1 disrupts D-loops formed upon invasion of Rad51-covered ssDNA into a homologous sequence [37] and *MPH1* overexpression from the *GAL1* promoter inhibits BIR [38]. Overexpression of *MPH1* in *cdc9-1* cells resulted in alleviation of telomere elongation (Figure 5C) consistent with the hypothesis that telomere elongation is BIR-dependent. Thus, the telomere elongation in *cdc9-1* is genetically dependent on core components of BIR machinery Rad51, Rad52, nPif1, Pol32 as well as on its regulators Mph1 and the PCSS complex.

### The DNA damage induced phosphorylation of nPif1 on TLSSAES is required for its role in BIR

**Figure 6:**
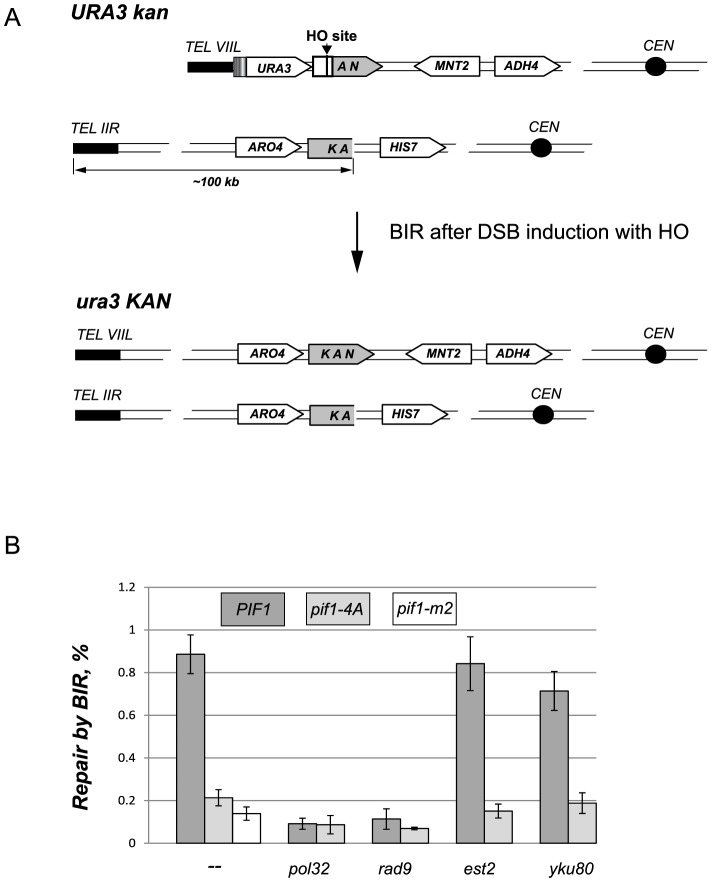
The DNA damage induced phosphorylation of nPif1 is required for BIR. (A) A schematic of the BIR assay. The 3′ fragment of *KAN* on *CHR VIIL* and the 5′ *KAN* fragment on *CHR IIR* possess 500 bp overlapping homologous sequence that can be used to initiate repair of the HO-induced DSB on *CHR VIIL* by BIR. The repair by BIR requires ∼100 kbp of the telomere proximal sequence on *CHR IIR* to be copied to *CHR VIIL*. The repair event also results in reconstitution of a functional *KAN* allele as well as in a loss of the *URA3* gene from the telomere *VIIL*. (B) The effect of *pif1-4A* mutant allele on BIR in different genetic backgrounds. The frequency of BIR was scored as a ratio of G418^R^ ura^−^ colonies to the total number of cells plated (see Materials and Methods). For each genotype, at least three independent experiments were used to calculate BIR frequency. Error bars represent standard deviations.

Since telomere elongation in response to *cdc9-1* was dependent on both BIR and nPif1 phosphorylation on TLSSAES, we tested if the nPif1 TLSSAES phosphorylation was required for BIR using a well-established system of galactose-inducible HO endonuclease expression to generate a DSB [39] next to two selectable markers, each located on either side of the break (see Figure 6A for details). The *KAN* selectable marker on the centromere-proximal side of the break was further separated into two non-functional fragments on *CHR VIIL* and *CHR IIR*, such that a functional allele of *KAN* would be restored only when BIR initiation at *CHR IIR* provided the template for repair of DSB on *CHR VIIL* (Figure 6A). Consistent with previously reported data [8], *PIF1* cells were more efficient at BIR than *pif1-m2*, and the *pif1-4A* mutants exhibited a loss of function similar to *pif1-m2* (Figure 6B). Both *pol32* and *rad9* abrogated *PIF1*-dependent BIR (Figure 6B), suggesting that nPif1 requires functional Polδ and DDR to promote BIR. Since *de novo* telomere addition is upregulated in *pif1-4A* cells [17] and thus might compete with BIR for DSBs, we tested whether *est2* or *yku80* mutations, which disrupt *de novo* telomere addition [18], affected BIR in *PIF1* and *pif1-4A* cells and found that the increased *de novo* telomere addition in *pif1-4A* did not impede BIR (Figure 6B). Therefore, the phosphorylation of nPif1 at the TLSSAES locus is required specifically for its role in BIR that is not affected by *de novo* telomere addition events that occur at DSBs.

### Longer telomeres in *cdc44-5* and *rrm3Δ* strains are further examples of BIR-dependent telomere elongation

Our hypothesis that *cdc9-1* cells suffer from broken replication forks as a result of replication passing through nicks is based on (i) the known role of Cdc9 in replication as a ligase of newly synthesized DNA strands [20], (ii) observed accumulation of unligated nascent DNA strands in the *cdc9-1* mutant [20], [33] which suggests nicks or single-stranded gaps, (iii) the established fact that replication through a nick results in a broken replication fork [34], and (iv) *RAD9*-dependent cell cycle arrest of *cdc9-1* mutants at the non-permissive temperature [20], [21] which can be explained by the existence of broken replication forks. If the hypothesis that broken replication forks repaired by BIR contribute to telomere elongation is correct, then there should be mutations in other components of replication machinery that also result in longer telomeres due to increased breakage of replication forks and activation of a DDR and BIR. Rrm3 is a non-essential DNA helicase required for replication through “hard-to-replicate” regions and the loss of *RRM3* results in a higher rate of fork breakage, constitutive activation of a DDR, and slight telomere elongation that is *PIF1*-dependent [40], [41]. *cdc44-5* is a temperature-sensitive mutant allele of Replication Factor C which operates as a PCNA clamp loader. At 30°C, *cdc44-5* mutants possess longer telomeres and accumulate cells in G2 [23]. We analyzed both *rrm3Δ* and *cdc44-5* for constitutive activation of a DDR and telomere elongation, and the possible dependence of the latter on Pol32 and nPif1. The DDR was constitutively activated in both *rrm3Δ* and *cdc44-5*, with more pronounced Rad53 phosphorylation observed in *cdc44-5* cells (Figure S4A). The slight telomere elongation in *rrm3Δ* was abolished by the *pif1-4A* mutation and there was no further increase in telomere length in *rrm3Δ pol32*Δ cells compared to either single mutant alone (Figure S4B). Combining *cdc44-5* with *pol32Δ* resulted in a synthetic lethality (Figure S4C) suggesting that BIR might be constitutively active in *cdc44-5* and required for cell viability (or that the combination of the two mutations could be too disruptive for conventional replication). The extensive telomere lengthening in *cdc44-5* cells was strongly suppressed by the *pif1-4A* mutation (Figure S4D). Therefore, both *rrm3Δ* and *cdc44-5* resemble *cdc9-1* in their constitutive activation of DDR and longer telomere phenotype and, like *cdc9-1*, telomere elongation in *rrm3Δ* cells is dependent on the BIR components nPif1 and Pol32 and the long telomere phenotype in *cdc44-5* is dependent on nPif1.

## Discussion

Here we report that the DNA damage-induced phosphorylation of nPif1, previously found to be critical for its role in inhibiting telomerase action at DSBs [17], is also required for functional BIR which in turn promotes telomere elongation.

The role of the replicative ligase Cdc9 in DNA replication is very well understood and its functional insufficiency in *cdc9-1* cells at the non-permissive temperature of 37°C leads to accumulation of unligated nascent DNA strands and *RAD9*-dependent cell cycle arrest [20], [21], [22], [33]. Growth of *cdc9-1* yeast at the semi-permissive temperature of 26°C predicts residual post-replication nicks: replication nicks are known to lead to DSBs as a result of broken replication forks [34]. We used the *cdc9-1* mutant allele to study how cells cope with such replication-coupled DSBs.

We found that the longer telomere length in *cdc9-1* cells stems from the activation of BIR and depended upon Rad51, Rad52, Pol32, and nPif1. The *cdc9-1* induced telomere elongation was also dependent on telomerase and therefore could not be attributed solely to recombination involving telomeric DNA. We postulate that the Rad51- and Rad52-dependence of telomere elongation in *cdc9-1* stems from constitutive activation of BIR, a homology-based mechanism of DSB repair, which in turn affects telomerase-dependent telomere elongation by either permitting a window of opportunity for telomerase at telomeres (i.e. when BIR forks reach chromosome ends) or via sequestration of nPif1 to BIR forks and removal of Pif1-dependent inhibition of telomerase access to telomeres.

**Figure 7:**
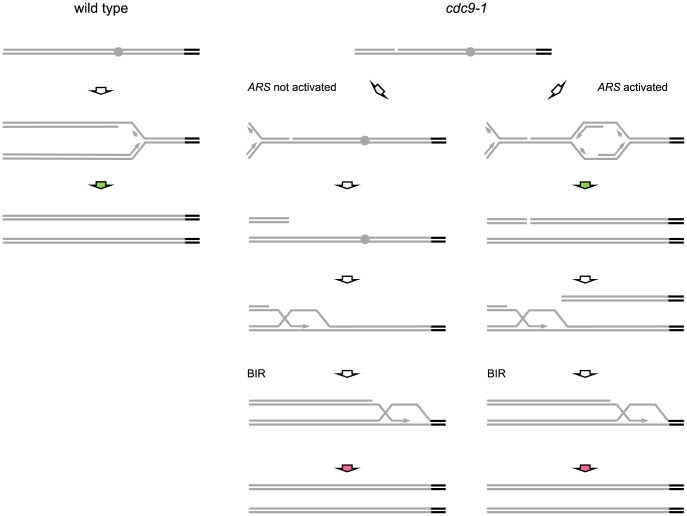
Possible mechanisms for replication-coupled telomere synthesis by telomerase in *CDC9* and *cdc9-1* cells. Grey bars and grey circles depict non-telomeric DNA and origins of replication within it respectively, whereas black bars correspond to telomeres. Green arrows represent telomerase activity coupled to conventional DNA replication whereas red arrows indicate telomerase action coordinated with BIR. In wild-type cells (see schematic at left), telomerase-dependent telomere elongation is coupled to conventional DNA replication of telomeric repeats (green arrow). In *cdc9-1* cells, nicks are present and replication through a nick would generate a broken replication fork with the remainder of the chromosome either not replicated, when there is no active *ARS* between the nick and a telomere (middle schematic), or replicated if replication is initiated within the region (schematic at right). The firing of replication origins (*ARS*) results in a passage of a conventional replication fork through the telomere (green arrow at right) and may be accompanied by telomere elongation by telomerase. However, the broken telomere-containing arm may be degraded/not involved in the repair of the broken DNA end on the other arm. Thus, in both cases, the repair in *cdc9-1* cells would proceed in a similar manner, via initiation of BIR that results in BIR-coupled action of telomerase at telomeres (red arrows). The telomeres in *cdc9-1* cells could be longer than in wild-type cells because the BIR-coupled telomerase activity (red arrows) is higher than when it is coordinated with conventional replication. Another possibility is that during BIR telomerase is provided with two windows of opportunity (the pathway on the right): one is when a conventional replication fork passes through a telomere (green arrow) and the other one is when the same telomere is replicated again by BIR (red arrow).

BIR might not be the primary pathway of repair for broken replication forks due to its increased mutagenesis rate [42], [43]. A broken replication fork is likely to be met by a fork coming from the opposite direction so that a two-ended DSB is generated and repaired via homologous recombination as suggested by Cortés-Ledesma and Aguilera [34]. However, BIR might be more common in yeast sub-telomeric regions containing genome duplications and repetitive sequences [44], particularly if no replication origin has been fired in the region between a broken fork and the telomere downstream. BIR shares many similarities with conventional replication [45], but the unique requirements for Pol32 and nPif1 [7], [8] as well as the use of conservative replication synthesis [5], [6] suggest structural and functional differences between the replication forks in conventional replication and BIR. Because telomerase-dependent telomere lengthening is coupled to the passage of replication forks through telomeres [12], [13] this coupling could be different during BIR and result in longer telomeres (Figure 7). In another scenario, two rounds of replication fork passage through a telomere within the same S-phase might be responsible for the longer telomeres in *cdc9-1* cells. First, a sub-telomeric origin is activated and a conventional replication fork passes through a telomere followed by a potential telomere elongation round. If a BIR fork moving towards telomere is established and if a previously replicated terminal fragment is not included in the repair, the BIR fork will pass through the same telomere creating another opportunity for telomerase to add more telomeric repeats. Therefore, two replication forks and potentially two rounds of telomerase action may take place and could account for the telomere elongation in *cdc9-1* cells. Either scenario would predict that BIR could affect telomere lengthening in *cis* but not in *trans*, i.e. only the telomeres passed by BIR forks would undergo additional lengthening. An alternative explanation of the telomere elongation in *cdc9-1* cells may stem from the effect of the DNA damage response on the regulation of telomerase by nPif1. In cells with no DNA damage, nPif1 inhibits telomerase at telomeres. However, during a DDR nPif1 localizes to the sites of damage and repair [8], [17]. The involvement of nPif1 in DSB repair may reduce its availability at all telomeres and result in a relief of the usual Pif1-dependent inhibition of telomerase at telomeres. This latter model would predict that the activation of BIR on a subset of chromosomes would affect all the telomeres to the same extent, i.e. the regulation would occur in *trans*.

### Compromised DNA replication affects telomere maintenance through DNA damage signaling

The DDR-dependence of telomere elongation in *cdc9-1* cells differed from the telomere elongation observed in *cdc17-1* cells, which is not DDR-dependent (Figure S1). We suggest that the effect of *cdc9-1* on telomere length is indirect, i.e. it is not due to uncoordinated chromosome replication and telomerase-dependent telomere lengthening. Instead, the DDR in *cdc9-1* cells is required for successful repair of broken replication forks via BIR that in turn causes additional telomere lengthening. We propose that this mechanism might explain previous reports that *rrm3* mutants, which suffer from replication fork pausing and breakage, also show constitutive activation of Rad53 and longer telomeres [40], [46]. A combination of constitutive damage signaling and telomere lengthening is also seen in *cdc44-5* cells. It is unknown whether the telomere lengthening in these mutants is DDR-dependent (*rrm3* cells require *RAD53* for their viability [46]), but the telomere elongation in both mutants was dependent on the DNA damage induced phosphorylation of nPif1. Pol32 was also required for longer telomeres in *rrm3* cells and the synthetic lethality between *cdc44-5* and *pol32Δ* suggests a possibility that BIR is critical for *cdc44-5* cell viability. In the genome-wide screens for *S. cerevisiae* deletion mutants causing telomere lengthening or shortening phenotypes, many genes that are relevant to DNA metabolism through replication, repair or chromatin structure have been reported [47], [48]. We suggest that like *cdc9-1*, the telomere length effects of some of these mutations may result from constitutive generation of DNA damage.

The dependency of the *cdc9-1* induced telomere lengthening on the DNA damage signaling is likely to be more complex than the requirement of the signaling kinases for nPif1 phosphorylation. The checkpoint activation is also necessary for the cell cycle arrest to ensure the completion of BIR [43], and therefore is also required for BIR fork passage through telomeres before the cells can enter mitosis. The signaling is also required for re-localization of the telomere bound proteins Sir3 and Ku in response to DNA damage [30]. This event might be relevant to the *cdc9-1* induced telomere lengthening as the latter was reduced in the *sir* mutants and abolished in the *yku70* and *yku80* backgrounds (Figure S2). The DNA damage induced changes in telomeric chromatin may affect telomerase access to telomeres so that telomerase is recruited to a larger number of telomeres and/or it is allowed longer time to elongate each telomere during S-phase, thereby increasing telomere length in cells with constitutive DNA damage.

### Implications of nPif1 regulation by DNA damage signaling pathway for the preservation of genomic integrity

Our findings suggest that the DDR-induced nPif1 phosphorylation does not only inhibit telomerase at DSBs as previously demonstrated, but it also plays a pivotal role in promoting BIR at broken replication forks and DSBs. Healing a broken DNA end by *de novo* telomere addition leads to global re-arrangements or partial loss of genetic material, thereby contributing to genome instability. Therefore nPif1 regulation directed at telomerase inhibition at DSBs [17] and facilitation of DSB repair via BIR (see Figure 6B) serve together to ensure genome repair and preservation. At the same time, the increased telomerase activity on telomeres as a result of an active BIR may have additional biological significance. For example, the telomere elongation in response to DNA damage could promote recruitment of additional telomere-associated chromatin factors (Rap1, Rif1, Sir proteins, etc.) that could in turn protect shorter telomeres against the DNA repair machinery activated in response to DNA damage [19]. In addition, the telomere lengthening via BIR could promote repair of critically short telomeres that have previously eroded and activated BIR in sub-telomeric regions. Since BIR leads to telomere lengthening, it may boost telomerase-dependent elongation of critically short telomeres involved in BIR. This mechanism could act in addition to the Tel1-mediated signaling which has been reported to increase telomerase recruitment on a shortened telomere [49], [50]. On the other hand, longer telomeres are harder to replicate as telomeric chromatin causes replication fork stalling [33], [41] and broken replication forks within telomeric repeats would result in truncated telomeres. Truncated telomeres after DSBs or as a result of active trimming [51] could therefore benefit from repair by telomerase activity. Thus, dynamic telomere length adjustments in response to changing extracellular and intracellular environment could have evolved to minimize genomic instability and maximize cell fitness and survival.

In summary, we uncovered that telomerase-dependent telomere elongation is modulated in response to DNA damage. Additional elongation occurs as a result of active BIR that requires Mec1-Rad53 dependent phosphorylation of nPif1. Therefore, nPif1 is a multifunctional regulator of telomere synthesis: it inhibits telomerase at telomeres in cells with no DNA damage, whereas in cells with DNA damage, its phosphorylation impairs telomerase action at DSBs and at the same time promotes telomere synthesis via BIR.

## Materials and Methods

*Yeast strains* are described in Table S1.

### Telomere length analysis (teloblots)

For genetic analysis of telomere length in different mutants, teloblot analysis was performed on a minimum of 3 tetrads, often 4-6 tetrads, that originated from at least 2 independently constructed parental strains. Relevant heterozygous diploids of each genotype were sporulated and progeny of at least four tetrads (at least two coming from each of the two diploids) were passaged for ∼80 generations at either 26°C (tetrads containing *cdc9-1* mutants) or 30°C (tetrads containing *rrm3* or *cdc44-5* mutants) on YPD agar (or YPGal agar if induction of *GAL1*- promoter was required), to equilibrate telomere length. Yeast genomic DNA was purified, digested with KpnI, and resolved on 0.85% w/v agarose gels. Southern blotting and hybridization were performed as described previously [33]. The random-primer (Prime-It II Kit, Stratagene) radiolabeled probe KL1 (recognizes the telomere proximal 650 bp of Y′ repeats) was used for hybridization in the KpnI experiments. In cases where slight variability in mutant behavior was observed, more than one tetrad (or clone) for the given combination of mutations was included in the corresponding figures (see Figure 3B for *cdc9-1 pif1-3A*, Figure 5B for *cdc9-1 psy3*, Figure S1 for *cdc17-1 rad53 sml1*, and Figure S4 for *cdc44-5 pif1-4A*).

### Immunoblotting

Yeast protein extracts were prepared using TCA precipitation [52]. Proteins were resolved on SDS-PAGE and transferred to PVDF membrane. Immunological detection was performed using ECL+ kit (GE Healthcare) according to the manufacturer's instructions. Mouse monoclonal α-myc 9E10 (Covance), goat polyclonal α-Rad53 yC-19 (Santa Cruz), and rabbit polyclonal α- VIDFYL(pT)LS(pS)AE (custom made, QCB) antibodies were used for Pif1-4myc, endogenous Rad53, and phosphorylated Pif1 detection respectively.

### Phosphatase treatments

Pif1-4myc was immunoprecipitated from cleared cell lysates at 4°C. Yeast cells were homogenized by bead beating in Lysis Buffer (25 mM HEPES (pH 7.5), 150 mM KCl, 1 mM EDTA, 1 mM EGTA, 0.1% v/v NP-40, 10% v/v glycerol) with phosphatase inhibitors (50 mM NaF, 50 mM sodium glycerophosphate) and protease inhibitors (Compete, EDTA-free protease inhibitors cocktail tablets, Roche). The lysates were cleared by centrifugation (14K rpm, 20′). Pif1-4Myc was immunoprecipitated by incubation of cleared lysates with 1∶150 dilution of α-myc antibody 9E10 (Covance) for 2 h followed by addition of Protein G agarose beads (Sigma) for an additional 2 h. Beads were washed 3×5′ in the Lysis Buffer and then rinsed in 1× phosphatase buffer supplied with either CIP or λ phosphatase (NEB). Phosphatase treatments were performed at room temperature in the corresponding buffers. Reactions were stopped by addition of SDS-PAGE sample buffer and 5′ boiling.

### Break induced replication assay

Cells were patched on YPRaffinose plates and grown overnight. Cells were re-suspended in YP broth to OD∼1. Serial dilutions were plated on YPD and YPGalactose agar. In 3 days, YPGalactose plates were replica-plated onto minimal media without uracil and on YPD+G418. The frequency of BIR was scored as a ratio of G418^R^ ura^−^ colonies to the total number of cells plated (represented by the number of colonies on YPD plates). At least two independently constructed strains for every genotype were used and at least three repeats for each genotype were performed to calculate standard deviations.

## Supporting Information

Figure S1 DNA damage signaling is not involved in *cdc17-1* telomere length phenotype. (A) Western blot analysis of Rad53 from WT, *cdc9-1*, and *cdc17-1* cells. (B) Telomere length analysis (teloblot) is of spore progeny grown for ∼80 generations at 26°C. Spores from the same tetrad (for *rad9*) or multiple spores with similar genotypes (for *rad53*) were analyzed. Unlike in *cdc9-1* cells at the semi-permissive temperature, neither *RAD9* nor *RAD53* is required for telomere lengthening in *cdc17-1* cells. DNA standards (in kbp) shown at left.
(EPS)

Figure S2 Genetic interactions between *cdc9-1* and genes encoding different components of telomeric chromatin. In each gel panel, telomere length analysis (by Southern hybridization) is shown for spore progeny from the same tetrad grown for ∼80 generations at 26°C. DNA standards (in kbp) shown at left.
(EPS)

Figure S3 Accumulation of late S/G2 cells in *cdc9-1* populations is not affected by either *pif1-4A* or *pol32Δ* mutation. FACS analysis of log-phase cells grown at 26°C. The *CDC9* alleles are shown on the left and the *PIF1* and *POL32* alleles are shown above the corresponding FACS profiles.
(EPS)

Figure S4 Analysis of telomere elongation in *rrm3Δ* and *cdc44-5* mutants. (A) Rad53 phosphorylation in *rrm3Δ* and *cdc44-5* analyzed by Western blotting; (B) Analysis of *rrm3Δ*-dependent telomere elongation in *pif1-4A* and *pol32Δ* backgrounds; (C) *cdc44-5* is synthetically lethal with *pol32Δ.* Progenies of 6 tetrads germinated for 72 h at 30°C (on the left) and their corresponding genotypes (on the right) shown. wt, 44, 32, and D represents spore genotypes *CDC44 POL32*, *cdc44-5 POL32*, *CDC44 pol32Δ*, and *cdc44-5 pol32Δ* respectively. (D) Analysis of *cdc44-5* dependent telomere lengthening in a *pif1-4A* background. DNA standards (in kbp) shown at left.
(EPS)

Table S1 *Saccharomyces cerevisiae* strains used in the study.
(DOCX)
