# Supplementary material for: Break-Induced Replication Requires DNA Damage-Induced Phosphorylation of Pif1 and Leads to Telomere Lengthening
Source: PLoS Genet. 2014 Oct 16;10(10):e1004679. doi: 10.1371/journal.pgen.1004679 (PMC4199488; doi:10.1371/journal.pgen.1004679)
Supplement: Table S1 — Saccharomyces cerevisiae strains used in the study. (DOCX) [file pgen.1004679.s005.docx]

Table S1 *Saccharomyces cerevisiae* strains used in the study

| Strain | Relevant genotype | Source/construction notes |
| --- | --- | --- |
|  | *Saccharomyces cerevisiaeA364a* |  |
| NK1 | *MATa ura3-52 trp1-289 leu2-3,112 bar1::LEU2* | Makovets & Blackburn, 2009 |
| NK3 | *MATα ura3-52 leu2-3,112 cdc9-1* | YJL347, Jochim Li Lab |
| NK5 | *MATα ura3-52 trp1-289 leu2-3,112 cdc17-1* | YJL353, Jochim Li Lab |
| NK18 | *MATa ura3-52 trp1-289 leu2-3,112 bar1::LEU2 pif1::KAN* | NK1 *pif1*::KAN |
| NK94 | *MATa ura3-52 trp1-289 leu2-3,112 bar1::LEU2 yku70::TRP1* | NK1 *yku70::TRP1* |
| NK96 | *MATa ura3-52 trp1-289 leu2-3,112 bar1::LEU2 yku80::TRP1* | NK1 *yku80::TRP1* |
| NK98 | *MATa ura3-52 trp1-289 leu2-3,112 bar1::LEU2 rif1::TRP1* | NK1 *rif1::TRP1* |
| NK100 | *MATa ura3-52 trp1-289 leu2-3,112 bar1::LEU2 rif2::TRP1* | NK1 *rif2::TRP1* |
| NK219 | *MATα ura3-52 trp1-289 leu2-3,112 bar1::LEU2* | Makovets & Blackburn, 2009 |
| NK227 | *MATa ura3-52 trp1-289 leu2-3,112 bar1::LEU2 rrm3::KAN* | Makovets et al., 2004 |
| NK398 | *MATa ura3-52 trp1-289 leu2-3,112 his3-del200* | YJL306, Jochim Li Lab |
| NK399 | *MATα ura3-52 trp1-289 leu2-3,112 his3-del200* | YJL307, Jochim Li Lab |
| NK402 | *MATα ura3-52 trp1-289 leu2-3,112 his3-del200 mec3::KAN* | NK399 *mec3::KAN* |
| NK404 | *MATα ura3-52 trp1-289 leu2-3,112 his3-del200 rad24::KAN* | NK399 *rad24::KAN* |
| NK406 | *MATα ura3-52 trp1-289 leu2-3,112 his3-del200 chk1::KAN* | NK399 *chk1::KAN* |
| NK408 | *MATα ura3-52 trp1-289 leu2-3,112 his3-del200 dun1::KAN* | NK399 *dun1::KAN* |
| NK410 | *MATα ura3-52 trp1-289 leu2-3,112 his3-del200 sml1::TRP1* | NK399 *sml1::TRP1* |
| NK414 | *MATα ura3-52 trp1-289 leu2-3,112 his3-del200 tel1::TRP1* | NK399 *tel1::TRP1* |
| NK415 | *MATα ura3-52 trp1-289 leu2-3,112 his3-del200 rad50::TRP1* | NK399 *rad50::TRP1* |
| NK423 | *MATα ura3-52 trp1-289 leu2-3,112 his3-del200 sml1::TRP1 mec1::KAN* | NK410 *mec1::KAN* |
| NK425 | *MATα ura3-52 trp1-289 leu2-3,112 his3-del200 sml1::TRP1 rad53::KAN* | NK410 *rad53::KAN* |
| NK427 | *MATa ura3-52 trp1-289 leu2-3,112 bar1::LEU2 cdc9-1* | NK3 x NK219 |
| NK429 | *MATα ura3-52 trp1-289 leu2-3,112 bar1::LEU2 cdc9-1* | NK3 x NK219 |
| NK439 | *MATα ura3-52 trp1-289 leu2-3,112 his3-del200 rad9::KAN* | NK399 *rad9::KAN* |
| NK490 | *MATα ura3-52 trp1-289 leu2-3,112 bar1::LEU2 dun1::KAN* | NK219 *dun1::KAN* |
| NK492 | *MATa/α CDC9/cdc9-1* | NK1 x NK429 |
| NK523 | *MATα ura3-52 trp1-289 leu2-3,112 bar1::LEU2 cdc9-1 yku70::TRP1* | NK94 x NK429 |
| NK524 | *MATα ura3-52 trp1-289 leu2-3,112 bar1::LEU2 cdc9-1 yku80::TRP1* | NK96 x NK429 |
| NK568 | *MATα ura3-52 trp1-289 leu2-3,112 bar1::LEU2 sir2::URA3* | NK219 *sir2::URA3* |
| NK570 | *MATα ura3-52 trp1-289 leu2-3,112 bar1::LEU2 sir3::KAN* | NK219 *sir3::KAN* |
| NK572 | *MATα ura3-52 trp1-289 leu2-3,112 bar1::LEU2 sir4::TRP1* | NK219 *sir4::TRP1* |
| NK828 | *MATa ura3-52 trp1-289 leu2-3,112 bar1::LEU2 pif1-m2* | Makovets & Blackburn, 2009 |
| NK857 | *MATa/α CDC9/cdc9-1 PIF1/pif1::pif1-4myc-KAN* | NK492::*pif1-4myc-KAN* |
| NK871 | *MATa ura3-52 trp1-289 leu2-3,112 bar1::LEU2 pif1::pif1-4myc-KAN* | NK857 sporulation |
| NK872 | *MATα ura3-52 trp1-289 leu2-3,112 bar1::LEU2 pif1::pif1-4myc-KAN cdc9-1* | NK857 sporulation |
| NK878 | *MATa ura3-52 trp1-289 leu2-3,112 bar1::LEU2 pif1-m2::URA3-pif1-m1* | Makovets & Blackburn, 2009 |
| NK882 | *MATa ura3-52 trp1-289 leu2-3,112 bar1::LEU2 pif1-m2::URA3-pif1-m1-3A* | Makovets & Blackburn, 2009 |
| NK918 | *MATa ura3-52 trp1-289 leu2-3,112 bar1::LEU2 pif1-m2::URA3-pif1-m1-4A* | Makovets & Blackburn, 2009 |
| NK920 | *MATa ura3-52 trp1-289 leu2-3,112 bar1::LEU2 pif1-m2::URA3-pif1-m1-4myc* | Makovets & Blackburn, 2009 |
| NK954 | *MATa ura3-52 trp1-289 leu2-3,112 bar1::LEU2 pif1-m2::URA3-pif1-m1-4myc cdc9-1* | NK429 x NK920 |
| NK967 | *MATa ura3-52 trp1-289 leu2-3,112 bar1::LEU2 pif1-m2::URA3-pif1-m1-4myc sml1::TRP1* | NK920 *sml1::TRP1*, note *PIF1* and *SML1* are linked |
| NK969 | *MATa ura3-52 trp1-289 leu2-3,112 bar1::LEU2 pif1-m2::URA3-pif1-m1-4myc sml1::TRP1 cdc9-1* | NK429 x NK967 |
| NK983 | *MATa ura3-52 trp1-289 leu2-3,112 bar1::LEU2 pif1-m2::URA3-pif1-m1-4A-4myc* | Makovets & Blackburn, 2009 |
| NK999 | *MATa ura3-52 trp1-289 leu2-3,112 bar1::LEU2 pif1-m2::URA3-pif1-m1-4A-4myc cdc9-1* | NK429 x NK483 |
| NK1003 | *MATa pif1-m2::URA3-pif1-m1-4myc tel1::TRP1* | NK414 x NK954 |
| NK1004 | *MATa pif1-m2::URA3-pif1-m1-4myc tel1::TRP1 cdc9-1* | NK414 x NK954 |
| NK1007 | *MATa pif1-m2::URA3-pif1-m1-4myc sml1::TRP1 mec1::KAN* | NK423 x NK969 |
| NK1008 | *MATa pif1-m2::URA3-pif1-m1-4myc sml1::TRP1 mec1::KAN cdc9-1* | NK423 x NK969 |
| NK1011 | *MATa pif1-m2::URA3-pif1-m1-4myc sml1::TRP1 rad53::KAN* | NK425 x NK969 |
| NK1012 | *MATa pif1-m2::URA3-pif1-m1-4myc sml1::TRP1 rad53::KAN cdc9-1* | NK425 x NK969 |
| NK1017 | *MATa pif1-m2::URA3-pif1-m1-4myc sml1::TRP1 dun1::KAN* | NK967 x NK429, *DUN1*/ *dun1::KAN* |
| NK1018 | *MATa pif1-m2::URA3-pif1-m1-4myc sml1::TRP1 dun1::KAN cdc9-1* | NK967 x NK429, *DUN1*/ *dun1::KAN* |
| NK3593 | *MATa/α CDC9/cdc9-1 POL32/pol32::HYG* | NK492 *POL32*/*pol32::HYG* |
| NK3725 | *MATa-inc ura3::NAT leu2::LEU2-Pgal-HO MNT2::kan::HOsite-URA3-STAR-TEL HIS7::kan* | Multiple steps. Note that the *kan* next to *MNT2* contains the 3’ part of *KAN-MX6* cassette beginning with GATGCTGATT whereas the *kan* next to *HIS7* carries the 5’ part of the cassette up to CATAAGCTTT. Both kan alleles are non-functional but contain 500 bp of overlapping sequence that provides homology to restore full *KAN-MX6.* |
| NK3728 | *MATa-inc ura3::NAT leu2::LEU2-Pgal-HO MNT2::kan::HOsite-URA3-STAR-TEL HIS7::kan pif1-m2* |  |
| NK3731 | *MATa-inc ura3::NAT leu2::LEU2-Pgal-HO MNT2::kan::HOsite-URA3-STAR-TEL HIS7::kan pif1-m2::TRP1-pif1-m1-4A* |  |
| NK3856 | *MATa-inc ura3::NAT leu2::LEU2-Pgal-HO MNT2::kan::HOsite-URA3-STAR-TEL HIS7::kan pol32::HYG* | NK3725 *pol32::HYG* |
| NK3858 | *MATa-inc ura3::NAT leu2::LEU2-Pgal-HO MNT2::kan::HOsite-URA3-STAR-TEL HIS7::kan pif1-m2::TRP1-pif1-m1-4A pol32::HYG* | NK3731 *pol32::HYG* |
| NK3860 | *MATa-inc ura3::NAT leu2::LEU2-Pgal-HO MNT2::kan::HOsite-URA3-STAR-TEL HIS7::kan rad9::HYG* | NK3725 *rad9::HYG* |
| NK3862 | *MATa-inc ura3::NAT leu2::LEU2-Pgal-HO MNT2::kan::HOsite-URA3-STAR-TEL HIS7::kan pif1-m2::TRP1-pif1-m1-4A rad9::HYG* | NK3731 *rad9::HYG* |
| NK3892 | *MATa-inc ura3::NAT leu2::LEU2-Pgal-HO MNT2::kan::HOsite-URA3-STAR-TEL HIS7::kan yku80::HYG* | NK3725 *yku80::HYG* |
| NK3896 | *MATa-inc ura3::NAT leu2::LEU2-Pgal-HO MNT2::kan::HOsite-URA3-STAR-TEL HIS7::kan pif1-m2::TRP1-pif1-m1-4A yku80::HYG* | NK3731 *yku80::HYG* |
| NK4286 | *MATa/α CDC9/cdc9-1 MPH1/mph1::TRP1-Pgal-MPH1* | NK492 *MPH1/mph1::TRP1-Pgal-MPH1* |
| NK4288 | *MATa/α CDC9/cdc9-1 PSY3/psy3::TRP1* | NK492 *PSY3/psy3::TRP1* |
|  |  |  |
|  | *Saccharomyces cerevisiae S288c* |  |
| SN1270 | *MATa MRC1* | Shivani Nautiyal |
| SN1273 | *MATa mrc1-AQ* | Shivani Nautiyal |
| CH1806 | *MATa CDC44_URA3 his4-539 lys2* | Adams & Holm, 1996 |
| CH1807 | *MATa cdc44-5_URA3 his4-539 lys2* | Adams & Holm, 1996 |
